# Data on the effect of NbC inoculants on the elastic and microstructural evolution of LBP-DED IN718

**DOI:** 10.1016/j.dib.2023.109299

**Published:** 2023-06-05

**Authors:** J.F.S. Markanday, M.A. Carpenter, R.P. Thompson, N.G. Jones, K.A. Christofidou, S.M. Fairclough, C.P. Heason, H.J. Stone

**Affiliations:** a University of Cambridge, Department of Materials Science & Metallurgy, 27 Charles Babbage Road, Cambridge, CB3 0FS, UK; b University of Cambridge, Department of Earth Sciences, Downing Street, Cambridge, CB2 3EQ, UK; c University of Sheffield, Department of Materials Science and Engineering, Mappin St, Sheffield City Centre, Sheffield S1 3JD, UK; d Rolls-Royce PLC, PO Box 31, Derby, DE24 8BJ, UK

**Keywords:** Additive manufacturing, Nickel, Superalloys, Anisotropy, Resonant ultrasound spectroscopy, Inoculants

## Abstract

The use of inoculants added to precursor powder is a method of influencing grain growth during fabrication. Niobium carbide (NbC) particles have been added to IN718 gas atomised powder for additive manufacturing via laser-blown-powder directed-energy-deposition (LBP-DED). The collected data in this study reveals the effects of the NbC particles on the grain structure, texture and elastic properties, and oxidative properties of LBP-DED IN718 in the As-DED and heat-treated conditions. The microstructure was investigated using X-ray diffraction (XRD), scanning electron microscopy (SEM) coupled with electron backscattered diffraction (EBSD), and transmission electron microscopy (TEM) coupled with energy dispersive X-ray spectroscopy (EDS). Resonant ultrasound spectroscopy (RUS) was used to measure the elastic properties and phase transitions during standard heat treatments. Thermogravimetric analysis (TGA) is used to probe the oxidative properties at 650°C.

**Specifications Table**

**Table:** 

Subject area	Additive Manufacturing
More specific subject area	Characterization of the microstructure and elastic properties of standard and NbC modified LBP-DED IN 718
Type of data	Figure and Table
How data was acquired	A phase analysis was carried out through XRD and TEM-EDS. XRD was completed using a Bruker D8 X-ray diffractometer and DSC was performed on a Netzsch 404 calorimeter with cylindrical specimens of a diameter of 5 mm and a thickness of 1.0 mm. TEM was performed on an FEI™ Tecnai Osiris TEM operated at 200 kV. A Zeiss Gemini SEM 450 equipped with an Oxford Instruments Symmetry EBSD detector and an Oxford Instruments X-MaxN 50 detector was used for microstructure and texture analysis. RUS was used to determine the elastic constants and calculate elastic properties. The oxidation performance was assessed using a Setaram Instruments Setsys Evolution TGA.
Data format	Raw
Description for Data Collection	Electro-discharge machining (EDM), performed in-house, was used to fabricate parallelepiped samples from LBP-DED IN718 builds. RUS was used to determine anisotropies in the As-DED and heat-treated states.
Data source location	University of Cambridge, Department of Materials Science and Metallurgy
Data accessibility	Included in this article and within the Apollo repository data, https://doi.org/10.17863/CAM.93482[1]
Related Research Article	J. F. S. Markanday, M. A. Carpenter, N. G. Jones, K. A. Christofidou, S. M. Fairclough, C. P. Heason & H. J. Stone, Effect of NbC Inoculants on the Elastic and Microstructural Effect of NbC Inoculants on the Elastic Properties and Microstructure of LBP-DED IN718, Materialia, Volume 27, March 2023, 101701, https://doi.org/10.1016/j.mtla.2023.101701

## Value of the Data

• These collected data provide useful insight to researchers on how the use of inoculants affect the microstructure and texture of an additively manufactured Ni-based superalloy.
• The NbC particles strongly influence the texture of LBP-DED IN718, significantly enhancing the Brass texture component (110) <211>. The ability to influence the texture of an additively manufactured Ni-based alloy is of interest to the field.
• The NbC inoculants are also found to refine the Laves phase and increase the volume fraction of the MC-type carbides in the microstructure. This increased the hardness of the alloy. No significant difference was found between the oxidative properties of standard LBP-DED IN718 and NbC inoculated IN718. Such data is useful in predicting the effect of compositional changes on additively manufactured IN718.

## Objective

These data were generated to support the manuscript: Effect of NbC Inoculants on the Elastic and Microstructural Effect of NbC Inoculants on the Elastic Properties and Microstructure of LBP-DED IN718. Additional details are provided about the specimens used in the main article as well as additional results that support the conclusions presented in the manuscript. The additional data include: XRD, TGA, SEM, SEM-EDX, SEM-EBSD and STEM-EDX analyses. These data are unique and provide additional value to the results presented in the main article.

## 1 Data Description

Information regarding the masses and dimensions of the IN718 and IN718-NbC parallelepiped samples used in this study have been provided in Table 1. The associated heat treatments protocols applied to the samples have been listed in Table 2. Microstructural and phase analysis were completed using a combination of XRD, DSC, SEM-EDS and STEM-EDS for samples in different heat-treated conditions. SEM analysis was used to assess the samples in the As-DED and heat-treatment A conditions. RUS analysis was used to measure the elastic constants at room temperature following the applied heat-treatment protocols. RUS was used to measure the change in elastic constants in-situ during the application of the standard heat-treatment protocols. From the data, elastic stiffness coefficients, anisotropy factors and acoustic loss data were obtained. The raw data collected using the XRD (text files), TGA (text files) and SEM (data and Aztec files) can be found in the associated Apollo repository [1].

**Table 1:** Dimensions and masses of the parallelepiped samples LBP-DED IN718 and IN718-NbC samples.

Sample	IN718 A	IN718 B	IN718-NbC A	IN718-NbC B
Dimension X (mm)	4.894	4.925	4.845	4.895
Dimension Y (mm)	3.853	3.829	3.860	3.878
Dimension Z (mm)	3.213	3.157	3.202	3.224
Mass (g)	0.4926	0.4812	0.4870	0.4935

The samples listed in Table 1 were subjected to the standard heat-treatment protocols listed in Table 2.

**Table 2:** Heat Treatment protocols applied to the parallelepiped IN718 and IN718-NbC samples fabricated via LBP-DED.

Heat Treatment	As-DED	A	B
Sample(s)	All	A Samples	B Samples
Temperature /°C	-	720 / 620	1100
Time / min	-	480 / 480	120
Cooling	-	Air	Air
Notes	Machined Condition	Two stage ppt treatment	ReX

## 2 Experimental Design, Materials and Methods

Monolithic builds were manufactured via the LBP-DED technique using standard gas atomised IN718 powder. The inoculated variant contained 1 percent by weight of 5-10 μm NbC particles added to the standard gas atomised IN718 powder. An internal EDM setup was used to fabricate parallelepiped samples from the LBP-DED builds. The dimensions and masses of the parallelepiped samples used have been given in Table 1.

**Fig. 1:**
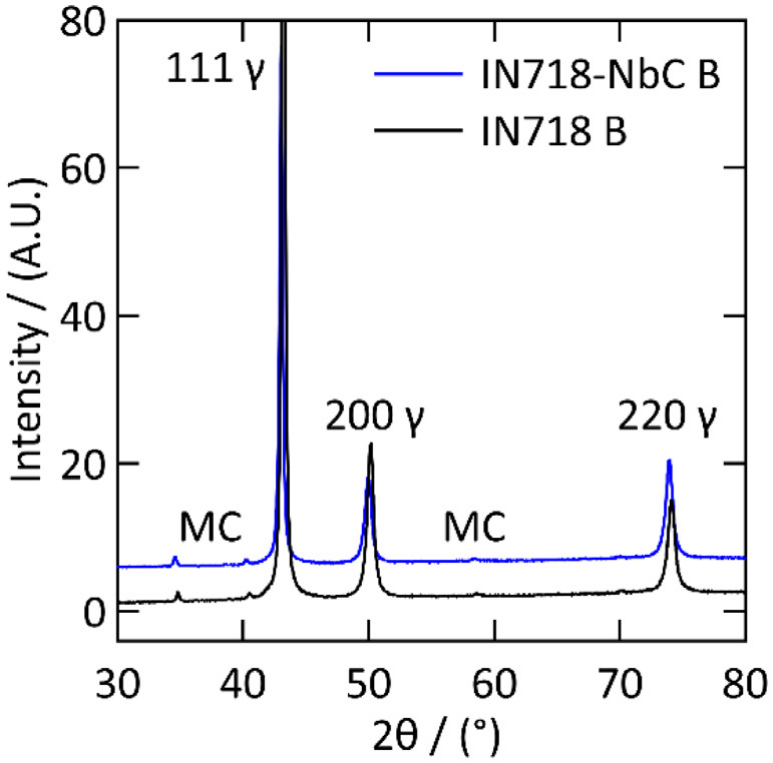
XRD patterns for LBP-DED IN718 and IN718-NbC samples in the heat treatment B condition. The reflections of the matrix γ phase and MC type carbide has been labelled.

Following application of the heat-treatment protocols the IN718 and IN718-NbC samples were analysed using XRD and SEM to assess the microstructure and phases. A Bruker D8 X-ray diffractometer equipped with a Cu-Kα source was used for the XRD analysis of the samples at 40 kV and 40 mA. Data were acquired from a 2θ range of 30° to 80° with a dwell time of 2.8 seconds and a step size of 0.05°. The XRD patterns for samples in the heat-treatment B condition are presented in Fig. 1. In both patterns the γ phase peaks have been identified and labelled, however the superlattice reflections of the γ′ and the γ′′ precipitates are not discernible. The peaks at 35° and 40.5° 2θ are present in both patterns and have been attributed to the MC-type carbide (111) and (200) reflections respectively. These peaks were present in both the As-DED and heat-treatment A conditions, along with Laves phase peaks at 37.5° and 45° 2θ, which are no longer present. The peak at 40.5° 2θ can also be attributed to the MC carbide phase.

DSC was performed on a Netzsch 404 calorimeter to investigate phase transitions in the IN718 and IN718-NbC samples. For heating and cooling a rate of 10°C/min was used with a 10 min isothermal hold between steps. A constant Argon flow rate of 50 mL/min was used during testing. The onset and termination of the identified peaks have been given in Table 3.

**Table 3:** Onset and termination point for phase events observed in the DSC analysis of IN718 and IN718-NbC. All temperatures are given in degrees Celsius.

Peak	IN718 Onset	IN718 Termination	IN718-NbC A Onset	IN718-NbC B Termination
Primary Melting	1219	1354	1219	1350
MC Carbide	1290	1296	1298	1309
Laves/γ′/γ′′ dissolution	1057	1177	1056	1181
γ′′/δ Precipitation	771	960	768	962
γ′ Precipitation	517	600	518	604

**Fig. 2:**
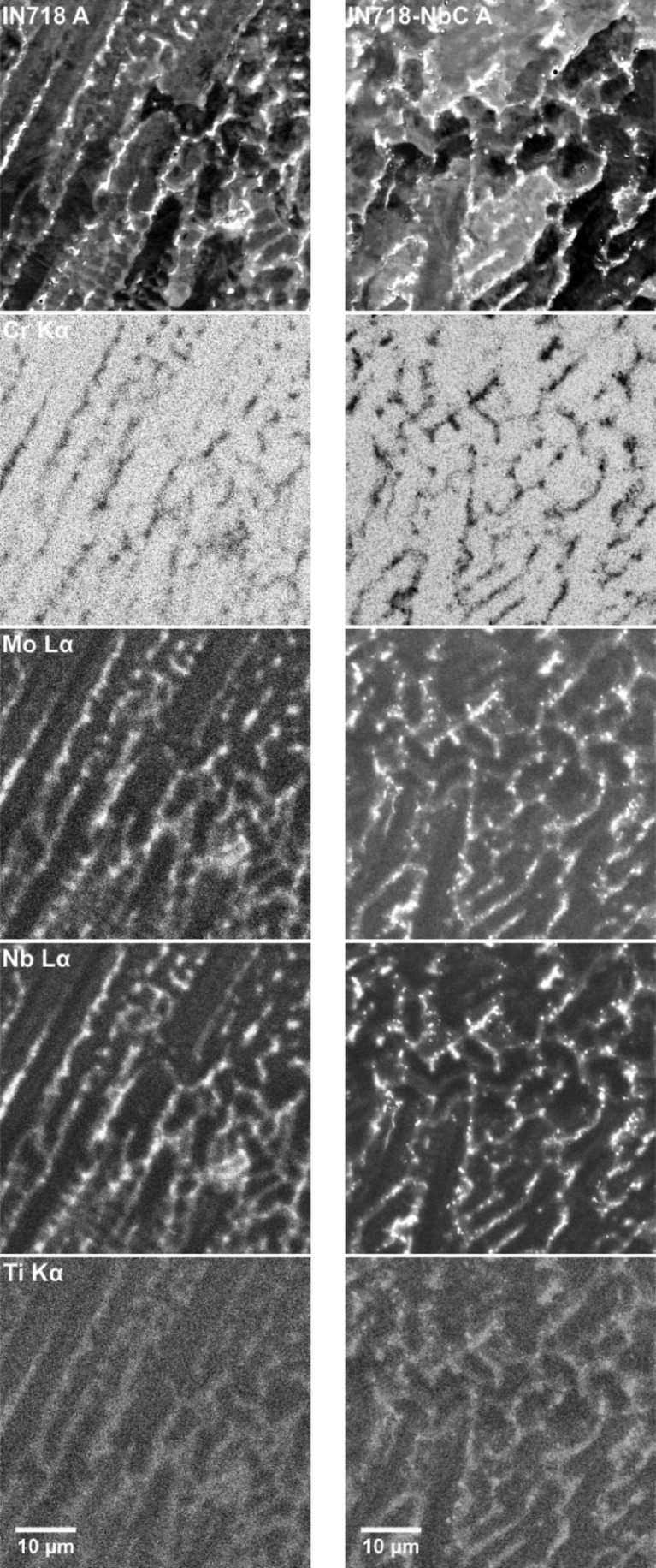
SEM-EDS analysis of IN718 (left) and IN718-NbC (right) samples in the heat treatment A condition. A backscattered electron image of the sample is given at the top, below this are elemental distribution maps for Cr, Mo, Nb, and Ti obtained by SEM-EDS.

**Fig. 3:**
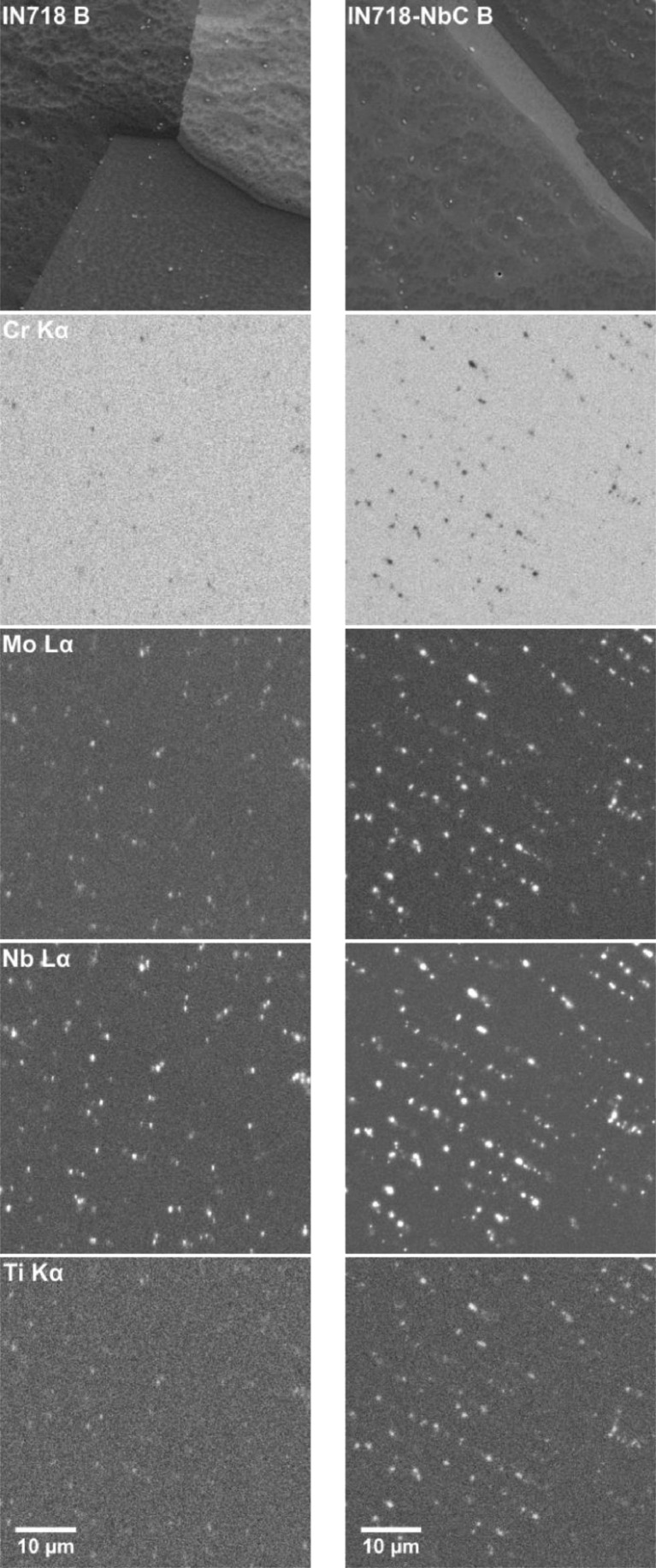
SEM-EDS analysis of IN718 (left) and IN718-NbC (right) samples in the heat treatment B condition. A backscattered electron image of the sample is given at the top, below this are elemental distribution maps for Cr, Mo, Nb, and Ti obtained by SEM-EDS.

An EDS analysis was performed using a Zeiss Gemini SEM 450 operated at 20 kV and equipped with an Oxford Instruments X-MaxN 50 detector. The SEM-EDS results for the IN718 and IN718-NbC samples in the heat treatment A and B condition have been presented in Fig. 2, Fig. 3 respectively. The results for the samples in the As-DED condition can be found in the original study. For the heat treatment A condition, significant segregation to the interdendritic regions is observed in both samples. The primary phase in this region is the Laves phase. For the IN718-NbC samples there is a higher volume fraction of spherical carbides and the Laves precipitates are more discrete. In the heat treatment B condition both samples display a recrystallised microstructure. The interdendritic precipitates have been dissolved and the spherical carbides are more prominent. There is noticeably higher volume fraction of the carbides in the IN718-NbC sample.

**Fig. 4:**
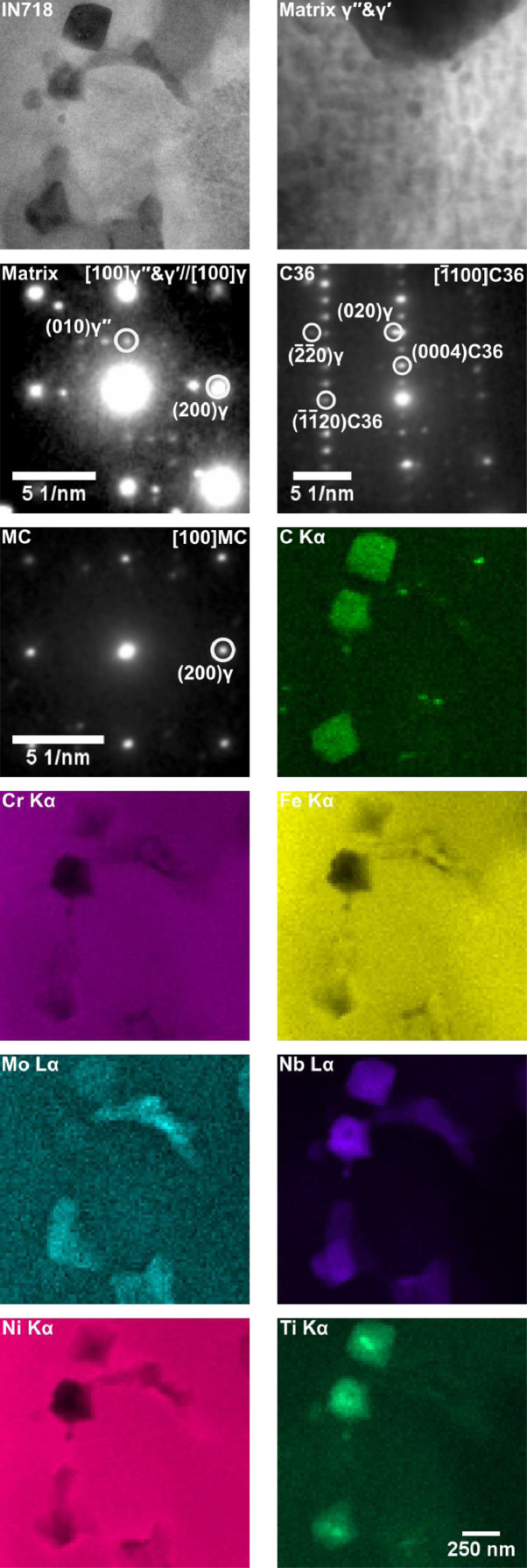
TEM analysis of LBP-DED IN718 in the heat treatment A condition. Annular dark field electron images are shown at the top, these are accompanied by selected area electron diffraction patterns for the matrix [001], MC carbide [100] and C36 Laves $\left[\bar{1}100\right]$. Below are elemental distribution maps determined by STEM-EDS from the field of view shown in the top left electron image.

A STEM-EDS analysis of the samples was carried out using an FEI™ Tecnai Osiris TEM operated at 200 kV. The results for the standard LBP-DED IN718 sample have been given in Fig. 4. The analysis for the IN718-NbC sample can be found in the original study. The analysis identified several phases within the γ matrix. These phases include the γʹ/γʹʹ, MC-type carbides and the Laves phase. The selected area diffraction patterns (SADPs) and EDS analysis confirm the presence of these phases. The details of the chemical analysis can be found in the original study. The morphology of the phases in both the IN718 and IN718-NbC samples are similar. There is only a significant difference in the compositions of the MC-type carbides, this is to be expected due to the addition of the NbC particles

**Fig. 5:**
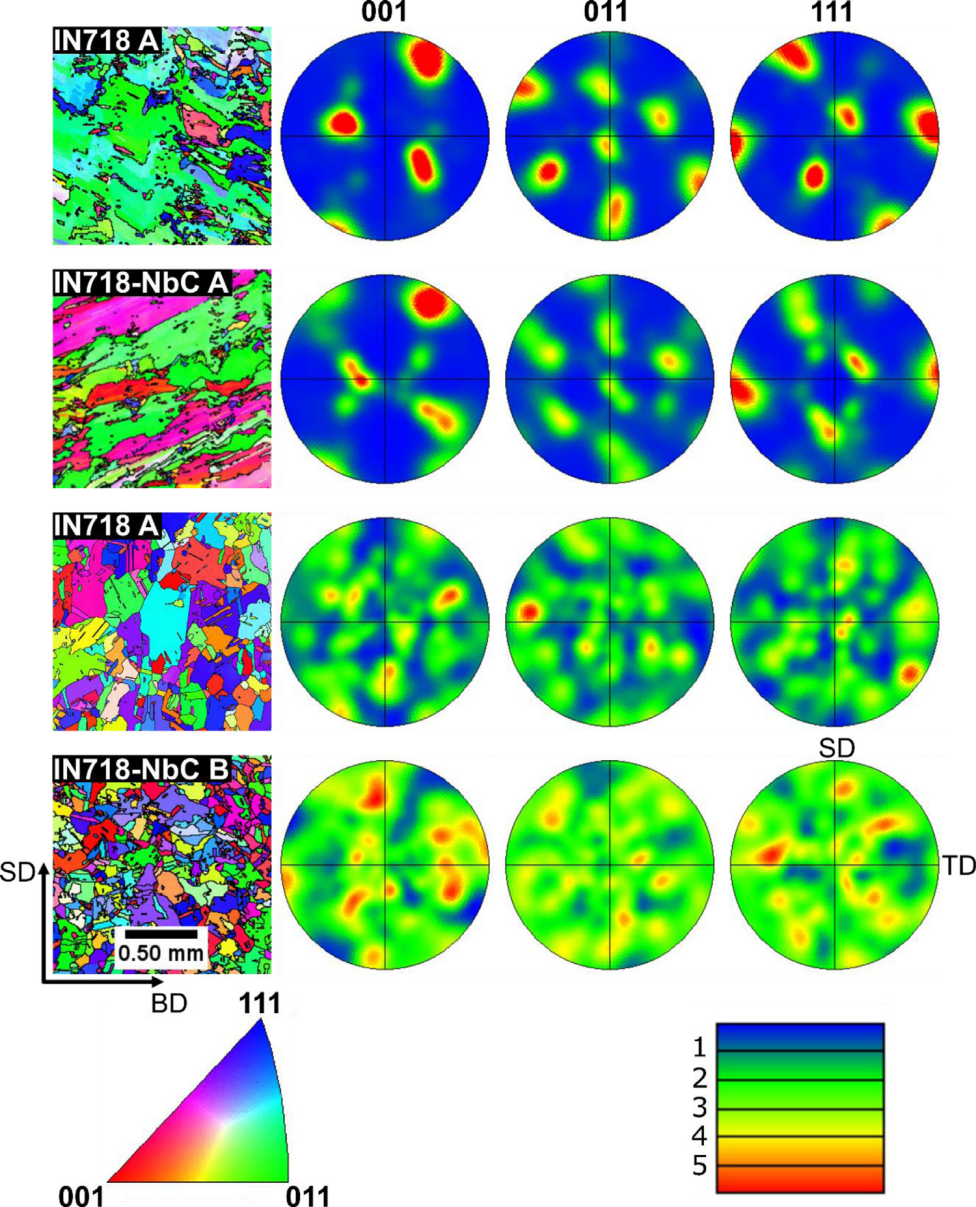
Inverse pole figure maps with respect to the build direction (BD) and scanning direction (SD) for the LBP-DED IN718 and IN718-NbC samples in the heat treatment A and B conditions (Left). Corresponding pole figures for {001}, {011} and {111} poles in the BD plane of the samples with the SD and transverse direction (TD) labelled .

A Zeiss Gemini SEM 450 equipped with an Oxford Instruments Symmetry EBSD detector was used for analysis of the texture of the IN718 and IN718-NbC samples. The orientation maps were acquired from areas of 3 mm^2^; 5° was chosen as the angular limit for cell/grain boundaries. For contouring of pole figures the parameters chosen were a half width of 15°, a cluster size of 5° and a multiple of uniform density (mud) of 6 mud for samples in A condition. The EBSD analysis for the IN718 and IN718-NbC samples in the heat treatment A and B conditions are given in Fig. 5. Additional results for samples in the As-DED condition can be found in the original study. For IN718 and IN718-NbC samples in the As-DED and heat treatment A condition a Brass component (110) <211> was identified as the primary texture. However, in the IN718-NbC samples the component was significantly enhanced giving rise to additional symmetry related spots in the pole figures. Following recrystallisation heat treatment B the samples were observed to have an equiaxed microstructure with no predominant texture. The recrystallised microstructure in the IN718-NbC sample was much finer due to the enhanced Zener pinning which arises due to the increased carbon content.

**Fig. 6:**
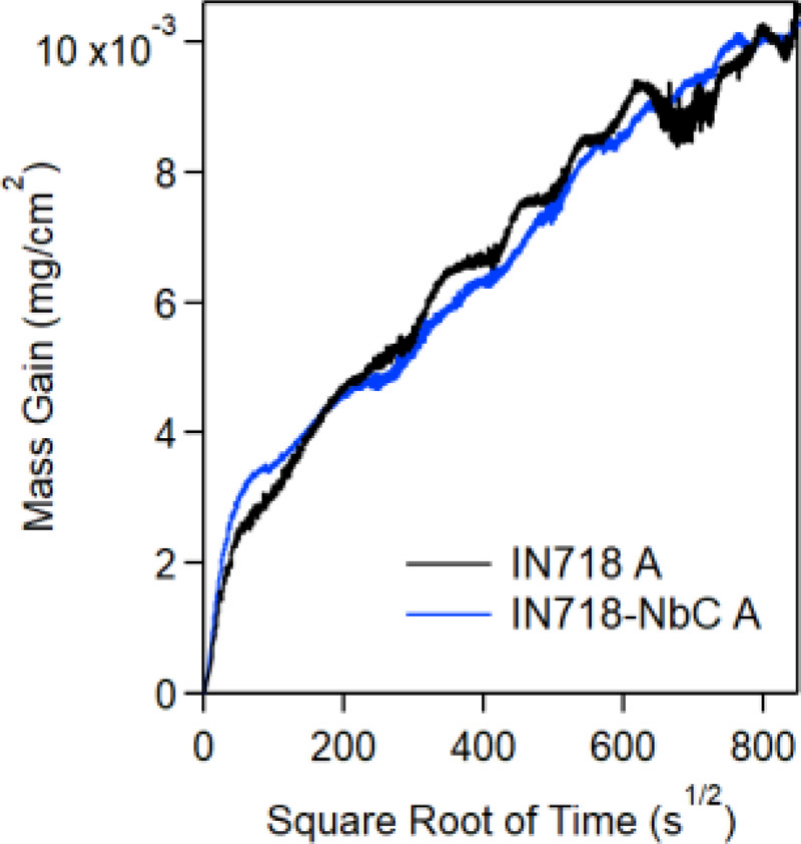
Mass gain during isothermal oxidation of LBP-DED IN718 and IN718-NbC samples during exposure at 650°C for 200 hours.

A TGA analysis was performed to assess the oxidation performance of the samples at 650°C for a 100-hour exposure. A Setaram Instruments Setsys Evolution TGA was used for the TGA analysis. Samples for TGA were in the heat treatment A condition and of the dimensions 20 × 10 × 1 mm. Samples were hung from a microbalance with a continuous stream of air flowing at 30 mL/min and a pressure of 1 bar. The mass gain data for the IN718 and IN718-NbC samples have been presented in Fig. 6. These data show that the samples have highly similar oxidation profiles. The mass gains recorded are extremely small, near the mass detection limit for the TGA. As such, undulations due to the temperature changes during the night and day have slightly affected the results. However, the final mass gain values are deemed to be valid.

The results from the RUS analysis have been given in Table 3, see original study for additional results. For RUS, spectra containing 50000 data points were collected over a frequency range of 100–1200 kHz. The full details of the experimental methods used can be found in the work of Migliori and Sarrao [2] and McKnight et al. [3,4]. The Wavemetrics IGOR Pro software package was used for analysis of RUS spectra with individual peak fitting completed using an asymmetric Lorentzian function. The elastic stiffness coefficients and elastic moduli were calculated for each LBP-DED IN718 parallelepiped sample using the open-source rectangular parallelepiped resonances (RPR) code [2]. Uncertainties in the elastic moduli were below 2.5 % in all cases.

Three terms A_100_, A_010_ and A_001_ Eq. 1–(3), were used to quantify the elastic anisotropy of the samples in the cubic shear-planes. A perfectly isotropic single crystal would have values of 1 for the three terms. The work of Ravindran et al. [5] was used in the derivation of these coefficients. The anisotropy coefficients for each sample have been given in Table 4.

(1) $$A_{100}=\frac{4C_{44}}{C_{22}+C_{33}-C_{23}}$$

(2) $$A_{010}=\frac{4C_{55}}{C_{11}+C_{33}-C_{13}}$$

(3) $$A_{001}=\frac{4C_{66}}{C_{11}+C_{22}-C_{12}}$$

**Table 4:** Elastic constants and properties for the LBP-DED IN718 parallelepiped samples in their respective heat treatment (HT) conditions. The sample has been labelled in brackets beneath the heat treatment (HT) condition. The elastic constants were calculated with sample dimensions for the RD, TD and BD representing the x (1), y (2) and z (3) axes, respectively.

										B	E	G	RMS
HT (Sample)	Stiffness Coefficients (GPa)									(GPa)	(GPa)	(GPa)	(%)
	c_11_	c_22_	c_33_	c_12_	c_13_	c_23_	c_44_	c_55_	c_66_				
IN718 A	308	300	280	113	116	142	90.0	68.9	68.1	181	207	79	0.40
IN718-NbC A	322	307	299	112	116	152	88.8	64.5	63.3	188	206	78	0.56

## Ethics Statements

No data was collected on human or animal subjects. In addition, no data was collected through social media platforms. All data collected complies with the ethical guidelines of the publisher.

## CRediT authorship contribution statement

**J.F.S. Markanday:** Conceptualization, Validation, Formal analysis, Investigation, Writing – original draft, Visualization. **M.A. Carpenter:** Methodology, Formal analysis, Resources, Investigation. **R.P. Thompson:** Formal analysis, Writing – review & editing. **N.G. Jones:** Formal analysis, Writing – review & editing. **K.A. Christofidou:** Investigation, Formal analysis, Writing – review & editing. **S.M. Fairclough:** Investigation, Formal analysis, Methodology. **C.P. Heason:** Resources, Supervision, Funding acquisition. **H.J. Stone:** Conceptualization, Writing – review & editing, Supervision, Funding acquisition.

## Declaration of Competing Interest

The authors declare the following financial interests/personal relationships which may be considered as potential competing interests: This work was funded by the EPSRC (through an iCase studentship) and by Rolls-Royce plc.

## Data Availability

Research Data on the Effect of NbC Inoculants on the Elastic and Microstructural Evolution of LBP-DED IN718 (Original data) (Apollo).
